# Tracing *Mycobacterium tuberculosis* transmission by whole genome sequencing in a high incidence setting: a retrospective population-based study in East Greenland

**DOI:** 10.1038/srep33180

**Published:** 2016-09-12

**Authors:** K. Bjorn-Mortensen, B. Soborg, A. Koch, K. Ladefoged, M. Merker, T. Lillebaek, A. B. Andersen, S. Niemann, T. A. Kohl

**Affiliations:** 1 Department of Epidemiology Research, Statens Serum Institut, Copenhagen, Denmark; 2 International Reference Laboratory of Mycobacteriology, Statens Serum Institut, Copenhagen, Denmark; 3 Greenland’s Center of Health Research, Nuuk, Greenland; 4 Institute of Clinical Medicine, University of Southern Denmark, Odense, Denmark; 5 Department of Internal Medicine, Queen Ingrid’s Hospital, Nuuk, Greenland; 6 Molecular and Experimental Mycobacteriology, Forschungszentrum Borstel, Leibniz-Zentrum für Medizin und Biowissenschaften, Borstel, Germany; 7 German Center for Infection Research (DZIF), partner site Borstel, Borstel, Germany; 8 Department of Infectious Diseases, Copenhagen University Hospital, Copenhagen, Denmark

## Abstract

In East Greenland, a dramatic increase of tuberculosis (TB) incidence has been observed in recent years. Classical genotyping suggests a genetically similar *Mycobacterium tuberculosis* (*Mtb*) strain population as cause, however, precise transmission patterns are unclear. We performed whole genome sequencing (WGS) of *Mtb* isolates from 98% of culture-positive TB cases through 21 years (n = 182) which revealed four genomic clusters of the Euro-American lineage (mainly sub-lineage 4.8 (n = 134)). The time to the most recent common ancestor of lineage 4.8 strains was found to be 100 years. This sub-lineage further diversified in the 1970s, and massively expanded in the 1990s, a period of lowered TB awareness in Greenland. Despite the low genetic strain diversity, WGS data revealed several recent short-term transmission events in line with the increasing incidence in the region. Thus, the isolated setting and the uniformity of circulating *Mtb* strains indicated that the majority of East Greenlandic TB cases originated from one or few strains introduced within the last century. Thereby, the study shows the consequences of even short interruptions in TB control efforts in previously TB high incidence areas and demonstrates the potential role of WGS in detecting ongoing micro epidemics, thus guiding public health efforts in the future.

In the first half of the 20^th^ century, Greenland experienced an extremely high incidence of tuberculosis (TB)^1^. By dedicated control efforts, the TB incidence rapidly declined and in the 1980s, TB was considered almost eliminated^1^,^2^,^3^,^4^. However, during the last decade, the population of the isolated East Greenland experienced a dramatic increase in TB incidence to approximately 1,000 cases per 100,000 inhabitants (Fig. 1) with more than 40% of the population being latently infected with *Mycobacterium tuberculosis* (*Mtb*)^5^. Especially high rates among children and young adults suggested recent transmission^5^,^6^,^7^. East Greenland is inhabited by only 3,500 people. They live in the main town Tasiilaq, five nearby settlements or in Ittoqqortoormiit 800 kilometres north of Tasiilaq. Contact between towns and settlements occurs on a regular basis by air or water transportation and many inhabitants move around in order to pursue jobs, education, or family relations. This mobility makes TB contact investigations complex, often involving inhabitants from multiple locations.

Available nationwide genotyping data document that almost all *Mtb* isolates from the region share very similar genotypes, hampering the use of most genotyping methods for analyses of transmission dynamics. However, recent studies have demonstrated that whole genome sequencing (WGS) of *Mtb* strains provides a higher resolution in delineating TB outbreaks, even in settings with highly similar strains^8^,^9^,^10^,^11^. Thus, WGS provides a unique opportunity to study transmission of *Mtb* in one of the world’s least populated regions with one of the highest TB incidences and with a low genetic diversity of circulating *Mtb* strains. By employing WGS, we reveal the long-term transmission dynamics and explore the potential implications for public health interventions in this TB high incidence setting.

## Results

From 1992 through 2012, 287 East Greenlandic TB cases were notified, of which 185 (64%) were *Mtb* culture-positive. For the remaining 102 cases, the laboratory register held information for cases diagnosed after 2000 (n = 91); four were confirmed by microscopy or PCR, 84 were culture- and microscopy negative and three had had no diagnostic samples examined at Statens Serum Insitut (SSI). Isolates from two patients could not be located, and library preparation failed for one isolate. Therefore, 182 (98%) culture-positive TB cases were included in our study (Table 1).

Mycobacterial interspersed repetitive units-variable number of tandem repeats (MIRU-VNTR) typing showed 10 unique strains and six clusters differentiated into two distinct complexes (Fig. 2). Two large clusters (1139–15 and 1144–17) comprised the majority of isolates (n = 134). WGS was successfully performed for all isolates, reaching an average coverage depth of the reference genome of at least 50 fold (range 51.6–437.4, median 93.5) and 98% of the reference genome was covered by at least one read. The median fraction of the reference genome fulfilling unambiguous criteria for variant detection was 98% (range 97–99).

**Figure 1:**
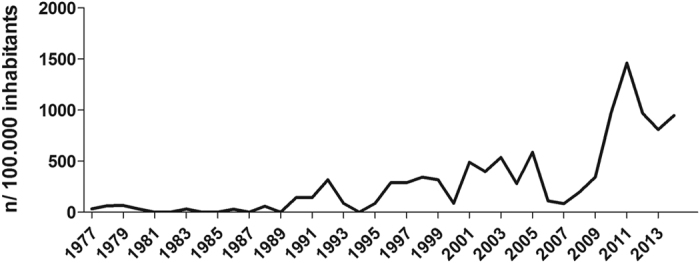
TB incidence in East Greenland. The number of notified TB cases in East Greenland per 100,000 inhabitants from 1977–2014. Reproduced with permission of the European Respiratory Society©: European Respiratory Journal Sep 2015, 46 (3) 865–869; doi: 10.1183/09031936.00012915.

**Figure 2:**
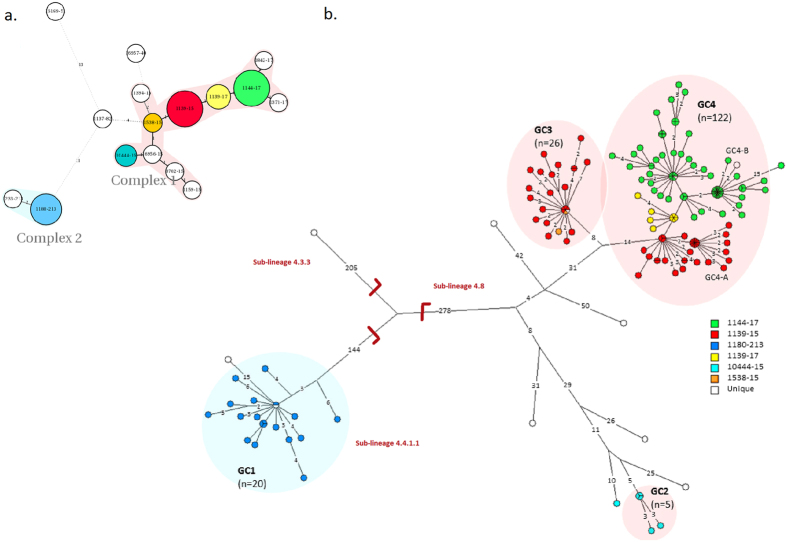
Genotypes of *M. tuberculosis* isolates from East Greenland. Genotypes of isolates from all (98%) culture-positive TB patients 1992–2012 from East Greenland (n = 182). (**a**) Minimum spanning tree based on MIRU-VNTR genotypes created with the MIRU-VNTRplus webpage. MIRU-VNTR clusters were defined as strains with identical genotyping patterns and clonal complexes by a maximum difference of two loci^33^. The colours represent five clusters and 10 unique strains grouped into two clonal complexes. (**b**) A maximum parsimony tree built from 1,385 single nucleotide polymorphism (SNP) positions. Branch lengths indicated by numbers representing distinct SNP positions. Branches of one SNP length are represented without numbers. Using a maximum distance of 12 SNPs to the nearest group member, all but seven isolates were grouped into four genomic clusters (GCs), which overall correlated with MIRU-VNTR genotypes and clonal complexes. Sub-lineages according to the schema proposed by Coll *et al*.^12^ are indicated on respective branches of the tree.

Four genomic clusters (GCs) as defined by a maximum distance of 12 SNPs and nine ungrouped strains were detected (Fig. 2). The majority of isolates belonged to GC4 (n = 122) and GC3 (n = 26). While all isolates from the large MIRU-VNTR cluster 1144–17 belonged to GC4, WGS divided the 1139–15 cluster into two clearly distinct groups differentiated by 22 single nucleotide polymorphisms (SNPs), with the larger group belonging to GC4 and the smaller group to GC3. Apart from this, GC1 (n = 20) and GC2 (n = 5) correlated with specific MIRU-VNTR genotypes. Interestingly, a few isolates with identical WGS genotype were split by MIRU-VNTR typing, thus, demonstrating the independent evolution of the markers. All isolates were also differentiated in major lineages and sub-lineages according to the schema proposed by Coll *et al*.^12^. All strains belonged to the Euro-American lineage (lineage 4), more specifically the 4.4.1.1 (GC1), 4.8 sub-lineage (GC2, GC3, and GC4), and one isolate from the 4.3.3 sub-lineage (Fig. 1).

**Figure 3:**
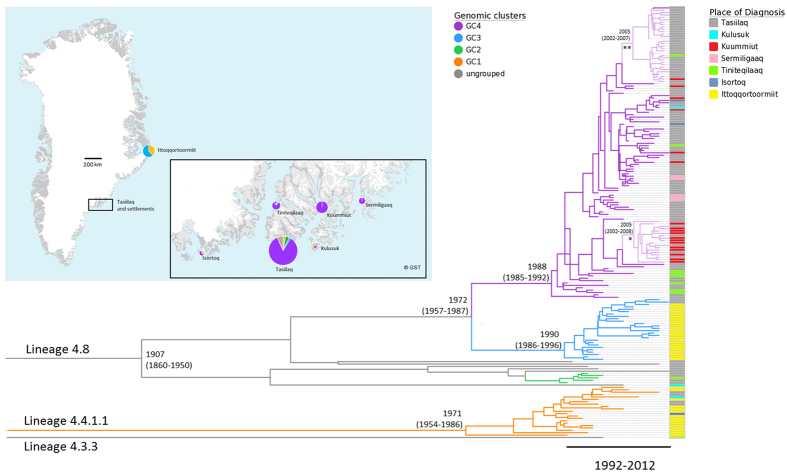
Bayesian coalescent analysis *M. tuberculosis* isolates from East Greenland. Geographical distribution, dating and classification of all (98%) culture-positive TB patients in East Greenland 1992–2012 (n = 182)). Emergence of the most recent common ancestor shown as calendar year with 95% highest posterior density interval. Coloured bars on right hand side represent place of diagnosis, while branch colour represent genomic clusters (GCs). For GC4, the lighter branch colour marks sub-clusters GC4-A (*) and GC4-B (**). Identified GCs and sub-clusters are supported by posterior above 0.99. Modified map from www.Nunagis.gl, Greenland 1:250.000, November 2015 with permission from The Agency for Data Supply and Efficiency.

**Figure 4:**
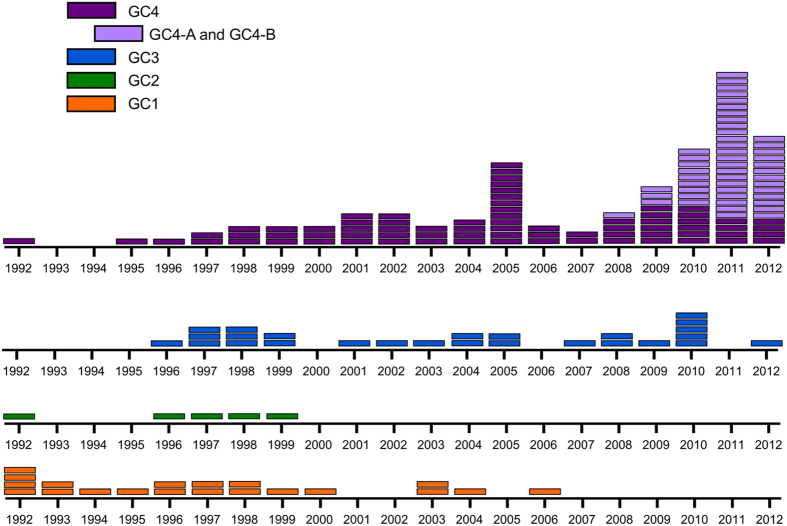
The year of diagnosis of culture-positive TB patients in East Greenland. Isolates from culture-positive TB patients 1992–2012 (n = 182) stratified into four genome clusters (GCs), with each rectangle representing an isolate from the respective year. For GC4, the lighter branch colour marks sub-clusters GC4-A and GC4-B.

The majority of isolates (n = 15/20) from the genetically distinct GC1 were from the remote town Ittoqqortoormiit, where they were probably introduced around 1971 (95% highest posterior density interval (HPD) 1954–1986) and continuously caused TB until 2000, after which only sporadic GC1 isolates appeared (Figs 3 and 4). In contrast, isolates from GC2, GC3, and GC4, in particular GC3 and GC4, constituted the majority of cases in the later part of the study period. The most recent common ancestor (MRCA) for these isolates (n = 160), including all strains from GC2, GC3, GC4, and seven ungrouped strains, dated back 105 years with a 95% HPD interval of 62–152 years (Fig. 3). GC3 and GC4 evolved around 1972 (95% HPD 1957–1987). Hereafter GC3 spread primarily in Ittoqqortoormiit after 1990 (95% HPD 1986–1996), while GC4 expanded in Tasiilaq and its five adjacent settlements after 1988 (95% HPD 1985–1992). The overall mutation rate estimated among all strains was 0.47 (95% HPD 0.37–0.64) SNPs per genome per year.

Isolates within GCs were closely connected, with more than half of isolates exhibiting a maximum distance of 5 SNPs to at least one other isolate. Yet, despite the close relationship of isolates, WGS suggested several sub-clusters within GC4 (Fig. 2). Two of these sub-clusters, GC4-A and GC4-B, are of special interest, since they both expanded after 2005 (95% HPD 2002–2008 and 2002–2007, respectively) (Fig. 3). The smallest of these two sub-clusters (GC4-A) comprised 18 highly similar isolates from TB cases diagnosed 2009–2012, including 11 isolates with identical genomes (Fig. 2). Twelve of these 18 TB cases were from the same settlement (Kuummiut). The remaining six cases were either reported as designated contacts to TB cases from Kuummiut (n = 2), or former residents of the settlement (n = 4). The larger sub-cluster GC4-B comprised 31 TB cases diagnosed from 2008–2012, of which 20 had identical genomes. The majority (n = 29/31) were diagnosed in the town of Tasiilaq (n = 29/31). Of the 30 cases diagnosed from 2010–2012, the majority (n = 26) were 16–23 years at time of diagnosis. Thus, they likely attended the same school and/or were part of the youth environment in Tasiilaq. Contact tracing links existed among 15 cases, which linked 4 of the 5 older cases to other cases in the sub-cluster. Contact tracing also confirmed possible transmission within other smaller sub-clusters of GC4, e.g. one sub-cluster including four young adults from smaller settlements, of whom three were brothers and/or have lived together at the boarding home in Tasiilaq. Thus, epidemiological data provided further evidence of transmission events within WGS suggested sub-clusters.

When analyzing the genome-based phylogeny dynamically over time, a simple algorithm that considers a minimum number of closely related isolates in one year was able to detect potential local outbreaks (Supplementary figure 1) likely associated with active transmission in the next year. That is, 79% (70/89) of identified putative outbreak isolates in 1992–2011 were associated with transmission events in the following year, compared to 15% (11/73) of non-outbreak isolates (Supplementary Figure S2).

## Discussion

This population-based study included 98% of all East Greenlandic culture-positive TB cases over two decades and allowed us to perform an in-depth analysis of the longitudinal *Mtb* transmission patterns in this unique, isolated, TB high incidence region. Our data indicated that the source of recent *Mtb* infections in East Greenland most likely dates back to the introduction of Euro-American strains within the last century, thereby indicating that recent TB in East Greenland is caused by reactivation of latent TB and subsequent transmission, even after decades of effective TB control measures^1^,^2^,^3^,^4^. Of the four genomic clusters detected, the closely related GC3 and GC4 were responsible for the majority of cases with a common ancestor that evolved 30–50 years ago and clonally expanded in the 1990s. In this challenging scenario for TB control, WGS detected several sub-clusters within the highly clonal strain population. Detection of these apparent micro epidemics by WGS is likely crucially important for outbreak characterization and a more targeted public health action in this setting.

Delineating *Mtb* transmission is difficult in high incidence settings with very similar strain populations and complex social interactions are challenging for TB control measures such as traditional contact tracing. Additionally, classical genotyping, e.g. MIRU-VNTR genotyping, is not efficient under these conditions. Here, WGS confirmed the clonality of *Mtb* strains in East Greenland. The majority of isolates were from sub-lineage 4.8, which was present in the region throughout the entire study period. This included isolates from the genomic clusters GC2, GC3 and GC4 and seven ungrouped isolates, all of which were within <100 SNPs of each other. The most common MIRU-VNTR genotypes within this sub-lineage (1139–15 and 1144–17) have only been reported sporadically from the rest of the Danish Kingdom. In contrast, isolates from GC1 correspond to a previously described Danish/Greenlandic cluster^13^,^14^, still being actively transmitted in Denmark and West Greenland, yet only sporadic cases appeared in East Greenland after 2000.

Dating analysis using Bayesian coalescent analysis suggested the introduction of the 4.8 sub-lineage into East Greenland some 100 years ago. Although we cannot exclude multiple introductions of this globally successful *Mtb* lineage into the region^15^, the limited genetic strain diversity and the isolated remote setting favours the hypothesis of indigenous evolution of these strain types in East Greenland. Our dating analysis nicely correlates with the foundation of a Danish colony in Tasiilaq in 1894, which promoted contact with Denmark and the rest of Greenland^16^ making the introduction of a strain from the Euro-American sub-lineage 4.8 thereafter plausible. An increasing TB incidence and *Mtb* infection prevalence described in East Greenland in the 1930s supports the hypothesis that *Mtb* was introduced a few years prior^17^. A Canadian study suggests an almost identical history of *Mtb* introduction into Nunavik, a high-incidence area in the Canadian arctic with even lower genetic strain diversity^10^,^11^.

Despite the close relationship of *Mtb* strains within the same genomic clusters, WGS allowed a detailed investigation of evolutionary history and longitudinal spread of the sub-lineage 4.8 population. Differentiation into the two largest genomic clusters (GC3 and GC4) presumably occurred in the 1970s. Since then, these two genomic clusters have spread to all East Greenlandic towns and settlements. In East Greenland, the 1970s was a time of great changes^18^. Many inhabitants moved from smaller settlements and contact between towns and settlements became easier with motorised boats and regular flights, potentially favouring the spread of *Mtb* in the following years. Yet, the most remarkable expansion of both genomic clusters occurred around 1990, and it is relevant to consider why this expansion occurred. In the 1980s, almost no TB was reported from East Greenland, or from Greenland in general^5^,^19^. TB was considered almost eliminated after decades of control efforts which led to a down-scaling of the TB control program. A lowered TB awareness in the 1980s and 1990s, could very likely have favoured conditions for *Mtb* expansion.

Our WGS analysis allowed a dissection of one MIRU-VNTR cluster (1139–15) into two distinct groups (Fig. 2). As described, one group caused TB in Ittoqqortoormiit (GC3), while the other caused TB in the rest of East Greenland (part of GC4). Ittoqqortoormiit, one of the most isolated towns in Greenland, was populated in 1925 by 87 persons from Tasiilaq^20^. However, the most recent common ancestor of GC3 and GC4 dates back to the beginning of the 1970s. Thus, the *Mtb* strains causing TB in Ittoqqortoormiit have been introduced into this town after the 1970s. It is striking that after the initial introduction of an ancestral GC3 strain into Ittoqqortoormiit, transmission of GC3 strains (and of GC1 strains as discussed above) has stayed remarkably confined^13^. Such a geographically confined *Mtb* transmission is also reported from the similar setting of Nunavik in Canada^11^. As suggested by the Canadian authors, this has important implications for future efforts to control TB in similar settings, since control measures should primarily be focused on the local community when an apparent TB outbreak is recognized.

In 2009, a sudden increase in TB incidence was noted in the small settlement of Kuummiut and subsequently in the rest of East Greenland. This led to speculations whether inhabitants from Kuummiut had brought TB to the rest of the region, supported by reports of the same MIRU-VNTR cluster (1139–15) causing TB in both Kuummiut and Ittoqqortoormiit and a very similar strain causing TB in Tasiilaq (1144–17). However, as discussed above, WGS shows a clear dissociation between 1139–15 strains from Ittoqqortoormiit and the rest of East Greenland. Likewise, the strains that caused TB in Kuummiut were only found in this settlement or in epidemiologically linked individuals (GC4-A). Interestingly, GC4-A strains and strains from a large sub-cluster causing TB in Tasiilaq (GC4-B) were the result of consecutive bacterial expansion in 2005. Thus, it is unlikely that the increasing TB incidence in Kuummiut after 2009 was directly related to the increasing TB incidence in the rest of East Greenland. In combination with the evidence that TB in East Greenland today is most likely caused by the same *Mtb* strain as in the early 20^th^ century, our study suggests that increasing TB incidence in recent years was not caused by one single coherent outbreak, but by several smaller outbreaks caused by genetically similar *Mtb* strains on multiple locations consecutively. Even though a causative interaction with lack of TB awareness in the 1990s is speculative, this study raises a difficult question: when it is safe to downscale TB control efforts in previously high incidence countries?

As mentioned earlier, delineating TB dynamics in high incidence settings is challenged by complex contact patterns and the possibility that very similar isolates spread consecutively. Several studies have applied WGS for outbreak investigations in TB low- and high incidence settings^8^,^9^,^21^,^22^, and the *Mtb* mutation rates of 0.3–0.5 SNPs per genome per year estimated in other studies^8^,^9^,^23^,^24^ are very similar to the overall mutation rate of 0.47 (95% HPD 0.37–0.64) SNPs per genome per year estimated in our analysis. However, while defining a minimum distance of 12 SNPs as a threshold for unlikely recent transmission and a maximum distance of 5 SNPs as the threshold for likely transmission is feasible in low incidence settings^9^, such a threshold is more difficult to define in high incidence settings^22^. Within the four genomic clusters detected in East Greenland, more than 50% of pairwise distances between isolates were within 5 SNPs. Thus, setting a specific lower threshold for likely transmission, e.g. to confirm or reject patient-to-patient transmission, is difficult^22^. The possibility of intra-patient evolution and mixed infections further complicate the deduction of direct links between cases^25^,^26^,^27^. Still, WGS identified smaller sub-clusters within the largest genomic cluster (GC4), in which transmission events were likely to have occurred. Since backwards mutation and SNP homoplasy are rare events in *Mtb* evolutionary history, our finding underlines that WGS enables a specific detection of outbreaks even in clonal strain populations in high incidence settings^26^,^28^,^29^. We tested this further by applying a simple SNP-based cut-off at a maximum of 3 SNPs, as proposed by Roetzer *et al*. as the maximum distance in human-to-human transmission^8^. Interestingly, this approach enabled the detection of “hotspot cases” associated with new cases appearing in the following year, thus likely identifying active transmission chains. This potentially allows for a WGS surveillance system using an SNP-based algorithm for automated outbreak detection and targeted public health action, if performing equally well in other settings and populations (Supplementary Figures 2 and 3).

The major strengths of this study are the completeness of included samples and the unique data linkage. However, all epidemiological analyses were made retrospectively, which potentially limited the accuracy of the data available. As an example, our analysis of the geographical distribution of samples were based on the place of diagnosis, which does not necessarily reflect a person’s place of living or the complex intraregional travel patterns. Another major limitation in our analysis are potentially missing TB cases. First, undetected TB cases in the area could have resulted in underestimating *Mtb* transmission. Secondly, *Mtb* DNA could only be obtained from TB cases with microbiologically confirmed *Mtb* infection, of which the percentage was low. Thus, only two thirds of all notified TB cases were included in the study. However, almost all of the non-included TB cases were culture- and microscopy negative and therefore not likely to cause *Mtb* transmission to any greater extent.

In conclusion, the combination of WGS and epidemiological data provided a unique understanding of the clonal *Mtb* expansion in a remote, TB high incidence setting. Since TB in East Greenland in recent years is most likely caused by an *Mtb* strain introduced into the region within the last century, and subsequently reactivated and expanded during a period of lowered TB awareness, the study provides evidence of the consequences of even small interruptions in the TB control efforts in previously TB high incidence areas. This is an important message for Greenland as well as for similar settings worldwide. WGS can detect sub-clusters caused by scattered cases in a high incidence setting, thus paving the way for WGS-based surveillance for targeted public health action.

## Methods

### Study design and population

We conducted a population-based study including all culture-positive TB cases diagnosed in East Greenland from 1992–2012. In Greenland, TB is mandatory notifiable to the National Board of Health, and contact tracing is performed routinely by local TB nurses. All notified TB cases with *Mtb* culture-positive samples were identified in the laboratory register at Statens Serum Institut which receives all diagnostic samples from Greenland. Culture-positive samples are stored at minus 80 °C.

Greenlandic citizens receive a unique personal identifier at birth through the Civil Registration System (CRS) enabling linkage across all public registers. Demographic data and contact tracing data were obtained from TB notifications, medical records or the CRS. Contact tracing data were only available from 2008–2012.

### Genotyping and sequencing

MIRU-VNTR 24-locus genotyping and DNA extraction from frozen isolates was performed as described elsewhere^30^,^31^. Libraries for WGS were prepared with the Nextera XT kit and run on Illumina next generation sequencing platforms (MiSeq, HiSeq 2500, Nextseq) as instructed by the manufacturer (Illumina, San Diego, CA). Reads were mapped to the genome of *Mtb* strain H37Rv (GenBank ID: NC_000962.3) with the exact alignment program SARUMAN^32^. SNPs with a minimum coverage of 10x and 75% allele frequency were called by customised Perl scripts. For the phylogenetic analyses, we excluded positions with SNPs in repetitive regions, in genes associated with drug resistance and neighbouring SNPs within a window of 12 base pairs. Only positions with a clear wild type or SNP allele call according to aforementioned thresholds in all isolates were combined for a concatenated SNP alignment (Supplementary Table S1). Raw data in the form of fastq files were submitted to the EMBL-EBI ENA sequence read archive (Supplementary Table S2).

### Phylogenetic analysis

A minimum spanning tree was created with the MIRU-VNTR*plus* database website. MIRU-VNTR clusters were defined as strains with identical genotyping patterns and clonal complexes by a maximum difference of two loci^33^. A maximum parsimony tree was calculated from 1,385 concatenated SNPs with Bionumerics^®^ version 7.5 (Supplementary Table S1) (Applied Math, Sint-Martens-Latem, Belgium). Phylogenetic lineages were determined according to lineage specific SNPs^12^. Strains were grouped into genomic clusters (GCs) defined by a maximum distance of 12 SNPs between one isolate and the nearest neighbour, as previously suggested as the minimum threshold of transmission^9^,^34^. Hence, genomic clusters were defined as groups of isolates between which transmission could have occurred. For all cluster-specific variants detected for the four genomic clusters see supplementary Table S3. Within GC4, sub-clusters were defined as groups of isolates clustering together in the phylogenetic tree. Whether these sub-clusters were actual clusters of *Mtb* transmission was explored by comparing WGS suggested links with contact tracing links and epidemiological data.

### Bayesian coalescent analysis

The concatenated SNP alignment was used for a Bayesian coalescent analysis using BEAST v.1.8.2^35^ (Supplementary file S4). The mean mutation rate was estimated from sampling dates with a tip-dating approach. Different demographic models were compared using a general time reversible (GTR) substitution model (strong support over HKY substitution model, log_10_ Bayes factor >10) with a discrete gamma distribution with four rate categories, a random starting tree, a lognormal relaxed clock, and assuming a uniform prior distribution and chain lengths of 150,000,000 with 10% burn in. Demographic models were compared with Tracer v.1.5 showing effective sample size (ESS) values in the hundreds and showed adequate mixing and convergence of the Markov chains. The likelihood of the different demographic models did not vary significantly (log_10_ Bayes factor <2), thus the coalescent constant size model (simplest model) were selected for dating analysis. A maximum clade credibility tree was created with TreeAnnotator v1.8.2 to identify diversification events giving the MRCA with 95% HPD.

### Ethical Considerations

The Committee for Scientific Research in Greenland approved the study (approval no. 2012-071304). The project was reported to and followed all instructions from the Danish Data Protection Agency.

## Additional Information

**How to cite this article**: Bjorn-Mortensen, K. *et al*. Tracing *Mycobacterium tuberculosis* transmission by whole genome sequencing in a high incidence setting: a retrospective population-based study in East Greenland. *Sci. Rep*. **6**, 33180; doi: 10.1038/srep33180 (2016).

## Supplementary Material

Supplementary Information

Supplementary Table S1

Supplementary Table S2

Supplementary Table S3

Supplementary Data

## Figures and Tables

**Table 1:** Demographic characteristics of TB cases in East Greenland.

	Culture-positive TB cases		Total TB cases	
	N	(%)	N	(%)
Total	**182**	(100)	**287**	(100)
Females	**88**	(48)	**134**	(47)
Males	**94**	(52)	**153**	(53)
Age at diagnosis				
<12 yr	**11**	(6)	**26**	(9)
13–19 yr	**47**	(26)	**80**	(28)
20–39 yr	**77**	(42)	**110**	(38)
>40 yr	**47**	(26)	**71**	(25)
TB type				
Recurrent	**25**	(14)	**37**	(13)
Pulmonary	**170**	(93)	**251**	(87)
Extrapulmonary	**42**	(23)	**73**	(25)
Pleural	**34**	(19)	**58**	(20)
Other^1^	**10**	(5)	**16**	(6)
Year of notification				
1992–1996	**18**	(10)	**25**	(9)
1997–2002	**42**	(23)	**67**	(23)
2003–2007	**36**	(20)	**61**	(21)
2008–2012	**86**	(47)	**134**	(47)
Place of notification				
Tasiilaq	**104**	(57)	**158**	(55)
Settlements	**39**	(21)	**70**	(24)
Ittoqqortoormiit	**39**	(21)	**59**	(21)
Laboratory				
Confirmed	**182**	(100)	**189**	(66)
Smear microscopy positive	**97**	(53)	**100**	(35)
Isoniazid monoresistance	**2**	(1)	**2**	(1)
Etambuthol monoresistance	**1**	(1)	**1**	(0)

Demographic characteristics of all cases of TB in East Greenland 1992–2012 (n = 287). The culture-positive samples (n = 182) included in the study are presented in the first column.
