# Supplementary material for: Tracing Mycobacterium tuberculosis transmission by whole genome sequencing in a high incidence setting: a retrospective population-based study in East Greenland
Source: Sci Rep. 2016 Sep 12;6:33180. doi: 10.1038/srep33180 (PMC5018808; doi:10.1038/srep33180)

SUPPLEMENTARY MATERIAL

Tracing *Mycobacterium tuberculosis* transmission by whole genome sequencing in a high incidence setting: a retrospective population-based study in East Greenland

Authors

K Bjorn-Mortensen*, M.D[1,2,3,4], B Soborg, M.D, ph.d.[1,] A Koch, M.D, ph.d[1], K Ladefoged, M.D, DMSc[5], M Merker, D.Sc[6], T Lillebaek**, M.D., DMSc[2], Prof. A B Andersen**, M.D., DMSc[4,7], Prof. S Niemann**, D.Sc [6,8], T A. Kohl**, D.Sc[6] . (**Equally contributing last authors)

Affiliations

[1]Department of Epidemiology Research, Statens Serum Institut, Copenhagen, Denmark, [2]International Reference Laboratory of Mycobacteriology, Statens Serum Institut, Copenhagen, Denmark , [3]Greenland's Center of Health Research, Nuuk, Greenland, [4]Institute of Clinical Medicine, University of Southern Denmark, Odense, Denmark, [5]Department of Internal Medicine, Queen Ingrid's Hospital, Nuuk, Greenland, [6]Molecular and Experimental Mycobacteriology, Forschungszentrum Borstel, Leibniz-Zentrum für Medizin und Biowissenschaften, Borstel, Germany, [7]Department of Infectious Diseases, Copenhagen University Hospital, Copenhagen, Denmark, [8]German Center for Infection Research (DZIF), partner site Borstel, Borstel, Germany

***Corresponding author**

Karen Bjorn-Mortensen

Department of Epidemiology Research/International Reference Laboratory of Mycobacteriology

Statens Serum Institut, Artillerivej 5, 2300 Copenhagen, Denmark

Email: kabm@ssi.dk, phone: +45 3268 8483

Supplementary figure S1: Yearly expansion of the TB outbreak in East Greenland.

Expansion of the maximum parsimony tree built from 1,385 SNP positions detected by WGS analysis of all culture-positive TB patients in East Greenland 1992-2012 (n=182). Isolates sampled in the respective year are marked in red, and detected outbreaks defined as 3 or more isolates within a maximum distance of 3 SNPS from each other appearing in the same year are encircled in orange.

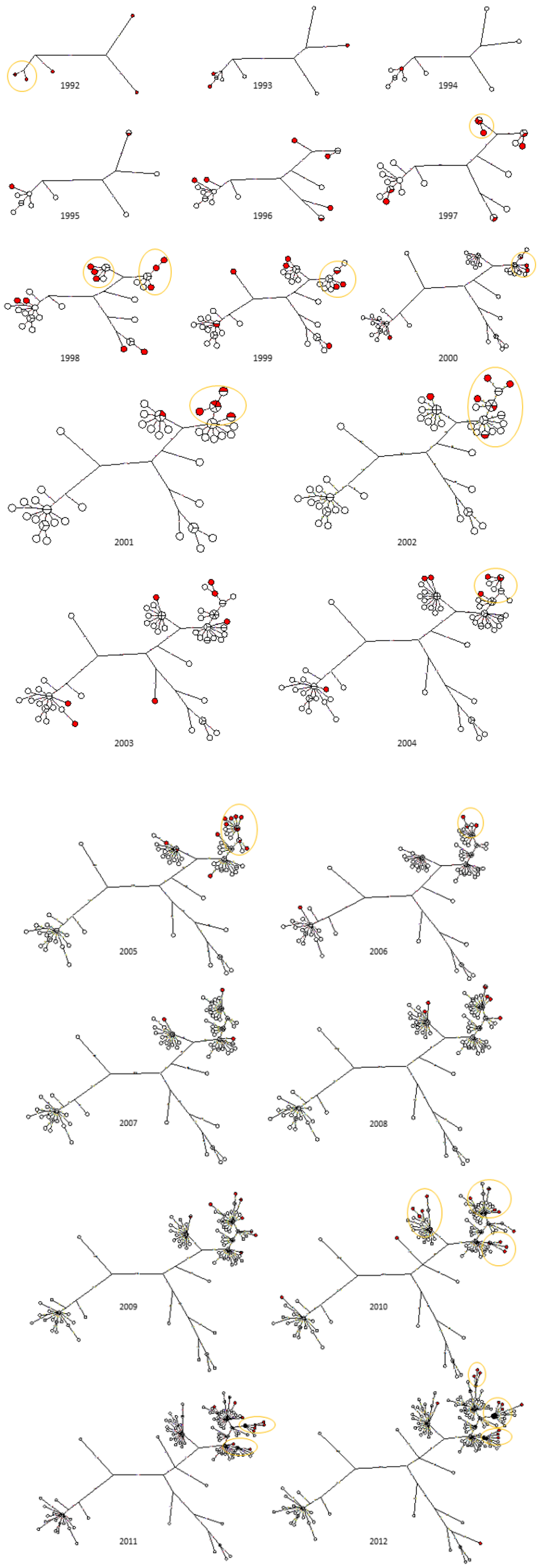

Supplementary figure S2: Experimental outbreak detection algorithm

This figure includes isolates from East Greenland from 1992-2012 and shows the number of new isolates within a maximum distance of 3 SNPs of either outbreak isolates (n=89) or non-outbreak isolates (n=73) and appearing in the following year. An outbreak isolate were defined as an isolate within a maximum distance of 3 SNPs of at least 2 other isolates in the same year. Hence, an outbreak were defined as 3 or more isolates within a maximum distance of 3 SNPS from each other appearing in the same year.

First, for each year, we determined the number of outbreak isolates in each outbreak (1992-2011). Then for these isolates as well as for the non-outbreak isolates, we determined how many new isolates that were detected within a maximum distance of 3 SNPs in the following year (1993-2012). If more than three new isolates appeared within 3 SNPs in the following year (per definition a new outbreak), the isolates from the first year was considered connected to an ongoing outbreak. The size of the bubbles reflects the number of isolates in each group.

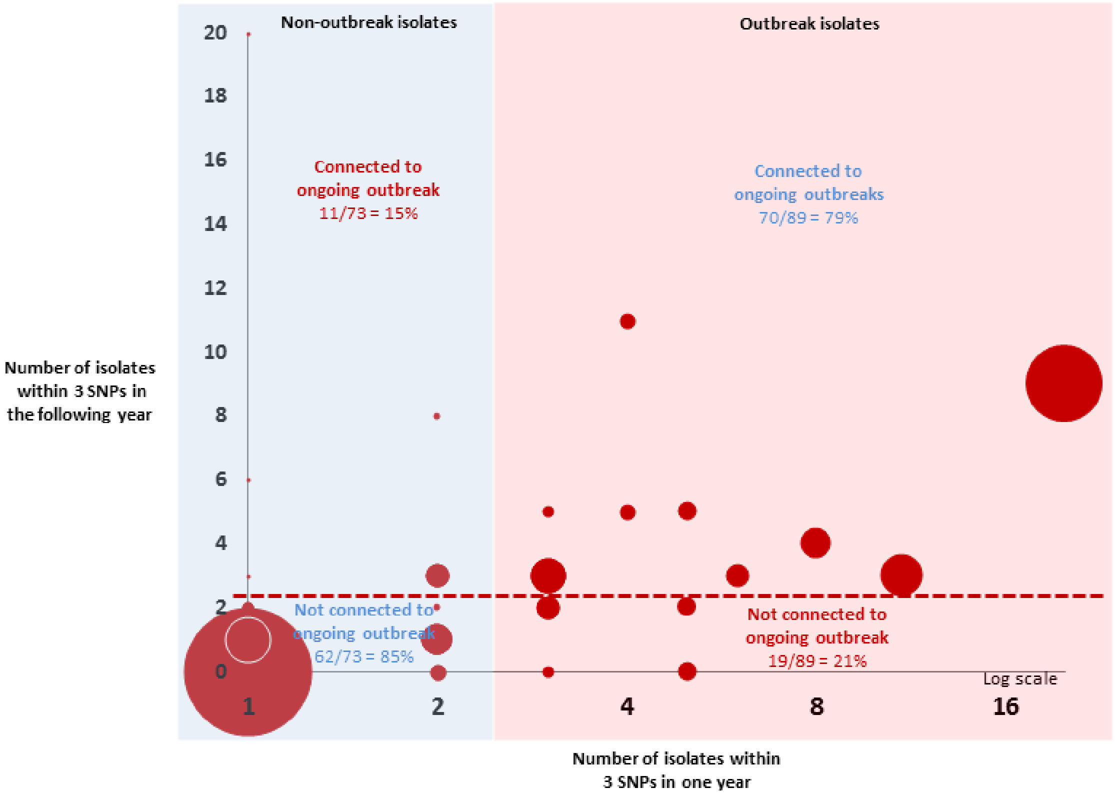

Supplement: Supplementary Information [file srep33180-s1.pdf]
